# Current Challenges and Long-Term Outcomes in Corneal Transplantation in Infectious Keratitis—A Systematic Review

**DOI:** 10.3390/jcm15020871

**Published:** 2026-01-21

**Authors:** Ancuța-Georgiana Onofrei, Alina Gabriela Gheorghe, Ana Maria Dascalu, Bogdan Mihai Cristea, Sinziana Istrate, Ana Maria Arghirescu, Dragos Serban, Corneliu Tudor, Paul Lorin Stoica, Marina-Ionela Nedea, Dan Dumitrescu

**Affiliations:** 1 Doctoral School, “Carol Davila” University of Medicine and Pharmacy, 020021 Bucharest, Romania; ancuta-georgiana.onofrei@drd.umfcd.ro (A.-G.O.); ana-maria.arghirescu@drd.umfcd.ro (A.M.A.); dragos.serban@umfcd.ro (D.S.); 2 BINE Ophthalmology Clinic, 020483 Bucharest, Romania; sinziana.istrate@umfcd.ro; 3 Department of Ophthalmology, Clinical Institute of Ophthalmological Emergencies “Prof. Dr. Mircea Olteanu”, 010464 Bucharest, Romania; alina.gheorghe.g@gmail.com; 4 Faculty of Medicine, “Carol Davila” University of Medicine and Pharmacy, 020021 Bucharest, Romania; corneliu.tudor@umfcd.ro (C.T.); stoica.paul91@gmail.com (P.L.S.); dan.dumitrescu@umfcd.ro (D.D.); 5 Ophthalmology Department, Emergency University Hospital, 050098 Bucharest, Romania; 6 Faculty of Pharmacy, “Carol Davila” University of Medicine and Pharmacy, 020021 Bucharest, Romania; marina.nedea@umfcd.ro

**Keywords:** infectious keratitis, fungal keratitis, bacterial keratitis, Acanthamoeba keratitis, therapeutic keratoplasty, penetrating keratoplasty, deep anterior lamellar keratoplasty, graft survival, visual recovery

## Abstract

**Background/Objectives:** Infectious keratitis remains a major cause of blindness worldwide, and many cases progress to therapeutic keratoplasty despite advances in antimicrobial therapy. This systematic review aims to evaluate the outcomes of therapeutic keratoplasty in microbial keratitis and examine factors influencing anatomical success, graft survival, and visual rehabilitation. **Methods:** A systematic review was conducted following PRISMA guidelines, including English-language studies, published between 2000 and 2025. Studies with ≥10 eyes and ≥6 months follow-up were included. Data on infection control, graft clarity, anatomical success, visual acuity, and complications were extracted. **Results:** Fourteen studies encompassing 1527 eyes were analyzed. TPK accounted for 89% of procedures; DALK was used selectively for anterior or mid-stromal infections. Overall infection control ranged from 69 to 100%, with globe preservation in 85–100% of cases. Bacterial keratitis had higher cure rates and graft clarity than fungal or Acanthamoeba keratitis. Larger grafts (>8 mm) and deep stromal involvement were associated with increased graft rejection and postoperative complications. DALK offered higher graft survival and lower immunologic risk when the endothelium was spared. Visual outcomes were generally limited, reflecting preoperative disease severity, timing of surgery, and postoperative immunomodulation constraints. Early surgical intervention improved anatomical outcomes in severe fungal keratitis. **Conclusions:** Therapeutic keratoplasty is an effective globe-preserving intervention in advanced microbial keratitis, but with limited functional outcomes. Further prospective studies are needed to refine surgical indications, postoperative management, and long-term functional results.

## 1. Introduction

Infectious keratitis is a significant cause of vision loss and blindness globally, especially in developing countries, despite the availability of increasingly effective antibiotics and antifungals. Without timely and proper treatment, the infection can cause extensive corneal infiltration, severe inflammation in the anterior chamber, perforation, and endophthalmitis, which may lead to enucleation [1]. In particularly severe cases of infectious keratitis, various therapeutic strategies are considered to prevent sight-threatening complications. In addition to topical and systemic use of antibiotics and antifungals, available interventions include intracameral or intrastromal injection, amniotic membrane transplantation, collagen cross-linking with photoactivated chromophore for infectious keratitis (PACK-CXL), and, in severe cases, therapeutic keratoplasty [2].

Among these approaches, therapeutic keratoplasty plays a central role in managing progressive infectious keratitis that is unresponsive to medical treatment [3]. Multiple surgical techniques are available, each with distinct indications, advantages, and limitations. Corneal transplantation can be broadly classified into full-thickness penetrating keratoplasty (PKP) and lamellar keratoplasty, which is further divided into anterior and posterior approaches (PLKs) [4].

Therapeutic penetrating keratoplasty (TPK) represents a critical surgical intervention for severe infectious keratitis that fails to respond to medical therapy or threatens corneal integrity. It is performed as a salvage procedure, aiming primarily to eradicate infection and maintain anatomical integrity, with visual rehabilitation as a secondary goal. When performed during the acute infectious stage, the procedure is associated with lower graft survival compared with elective transplantation in quiet, noninflamed eyes; therefore, surgical indications are generally highly selective. In clinical practice, TPK is generally indicated for large ulcers involving most of the corneal surface at presentation, for lesions progressing toward the limbus despite treatment, and in cases with descemetocele or frank perforation threatening globe integrity. Reported success rates of therapeutic keratoplasty vary widely, influenced by the virulence of the pathogen, the severity and extent of preexisting keratitis, the degree of ocular surface inflammation, the quality of prior medical treatment, and the surgical technique employed [5].

Among lamellar transplantation techniques, deep anterior lamellar keratoplasty (DALK) has emerged as the preferred approach in infectious keratitis. It has been reported to be effective in selected cases of fungal, bacterial, and Acanthamoeba keratitis, confined to the stroma, and refractory to intensive antimicrobial therapy. Current evidence supports performing the procedure earlier in the disease course, rather than reserving penetrating keratoplasty for the late, advanced phases of infection. A potential drawback of performing lamellar keratoplasty in this context is that infection eradication may be incomplete, occasionally leading to recurrence within the graft. Nevertheless, the procedure offers important advantages, notably a reduced risk of graft rejection and failure due to preservation of the recipient endothelium, as well as a lower likelihood of secondary endophthalmitis since the anterior chamber is not entered during surgery [6].

Despite advances in antimicrobial therapy and surgical techniques, several important clinical controversies persist in the management of infectious keratitis requiring corneal transplantation. Optimal timing of surgical intervention remains debated, as early therapeutic keratoplasty may improve infection control, but is associated with higher risks of graft failure and postoperative complications, whereas delayed surgery may allow disease progression and irreversible structural damage. Similarly, the choice between PKP and lamellar approaches, particularly DALK, remains challenging in the setting of active infection, where the extent of stromal and endothelial involvement is often difficult to determine with certainty [7,8]. In addition, postoperative management strategies, especially the timing and intensity of corticosteroid therapy, continue to represent a critical dilemma, balancing the prevention of graft rejection against the risk of infection recurrence, as highlighted in the studies discussed below.

Although fungal, bacterial, and Acanthamoeba keratitis differ substantially in terms of pathophysiology, microbiological characteristics, and medical treatment, they converge in the context of advanced disease requiring corneal transplantation. However, published data are largely from single-center retrospective studies or small case series, limiting generalizability. Moreover, there is no comprehensive synthesis of evidence comparing surgical approaches, graft types, and outcomes across different pathogens.

This systematic review aims to consolidate the current evidence on therapeutic keratoplasty for microbial keratitis, with a specific interest in optimal surgical strategies, graft selection, and timing. It also seeks to highlight knowledge gaps for future research, particularly in resource-limited settings where donor tissue availability and surgical expertise may constrain practice.

## 2. Methods

Given the clinical importance of this topic, we conducted a systematic review of the available literature to summarize the role of corneal transplantation in the management of microbial keratitis, with a focus on postoperative outcomes in terms of anatomic and visual rehabilitation.

A comprehensive literature search was performed across multiple electronic databases, including PubMed, Scopus, Web of Science, Springer Nature, and Science Direct, following Preferred Reporting Items for Systematic Reviews and Meta-Analysis (PRISMA) guidelines [9], by the keywords (“therapeutic keratoplasty” OR “penetrating keratoplasty” OR “DALK” OR “patch graft”) AND (“microbial keratitis” OR “infectious keratitis” OR “fungal keratitis” OR “bacterial keratitis” OR “Acanthamoeba keratitis”) AND (“outcome” OR “graft survival” OR “recurrence” OR “anatomical success”). All articles in the English language, published between 2000 and 2025, were screened for eligibility.

### 2.1. Study Selection

Articles were considered eligible if they addressed active infectious keratitis, including fungal, bacterial, Acanthamoeba, or mixed infections, for which corneal transplantation, either penetrating or lamellar, was part of the therapeutic strategy. Prospective and retrospective studies, including at least 10 eyes, with a follow-up period of a minimum of 6 months after surgery, reporting outcomes including anatomical integrity of the eye, infection control, graft clarity, and visual acuity were analyzed in the review.

The PICOS (Population, Intervention, Comparison, Outcome, and Study) strategy was employed to identify eligible studies to be included in the review:

P = Microbial keratitis patients;
I = Therapeutic keratoplasty (including PK/DALK/tectonic patch);
C = PK vs. DALK, if available in the study;
O = the main outcome was considered the anatomical success at the end of follow-up period; other secondary outcomes were documented, when available—final visual acuity; infection recurrence; and complications;
S = retrospective, prospective, and controlled trials were included.

Non-peer-reviewed materials, editorials, experimental studies, case-reports, and limited case-series were excluded. Studies including optical keratoplasty, microbial keratitis following corneal transplant, or non-infectious corneal ulcers requiring emergency surgery were not included in the analysis.

### 2.2. Data Collection and Quality Appraisal of the Studies Included in the Review

**Figure 1:**
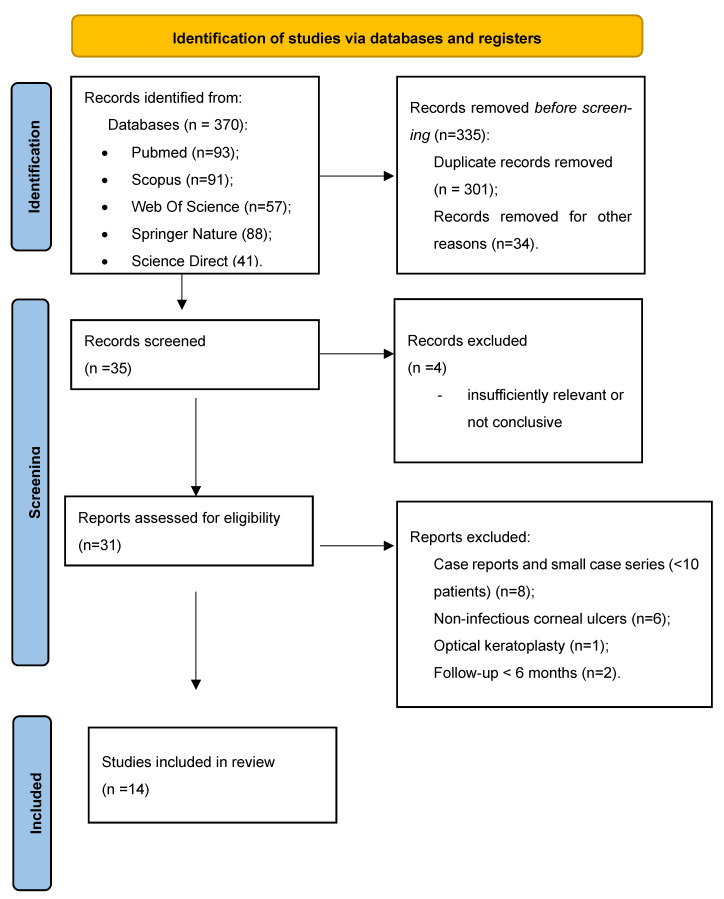
PRISMA flowchart for the studies included in the review.

The screening strategy was resumed in the PRISMA flowchart (Figure 1). The selection of the studies included in the analysis was performed by 2 independent researchers, with experience in corneal transplantation techniques. Any disagreement was solved by discussion.

The quality appraisal of the studies was performed using the Joanna Briggs Institute (JBI) Critical Appraisal Checklist for single-arm studies including >=10 eyes, and Newcastle–Ottawa Scale (NOS) for comparative cohort studies [10,11]. In the Supplementary Materials you can find the PRISMA checklist. Only studies graded as 7 or above were included in the qualitative analysis (Table 1).

Most included studies were retrospective case series of moderate to high methodological quality [12,14,15,16,17,19,20,21,22,23]. Higher quality was observed in larger cohorts and comparative studies [13,18,24,25], while common limitations included retrospective design, lack of consecutive recruitment, and heterogeneous outcome reporting.

All studies provided comprehensive information regarding etiology, perioperative management, and surgical technique. The outcomes were reported in terms of anatomical restoration, graft transparency, and visual acuity at the end of the follow-up period, as well as the associated complications. However, the comparability was limited due to the differences encountered in terms of the causative pathogen, timing, surgical procedure, and follow-up period, which may be a potential source of bias. Moreover, while some authors in the study selected only the cases with confirmed microbiologic etiology [12], others reported a lack of pathogen identification varying from 25 to 70% [14,17,18]. Thus, only a qualitative analysis could be performed.

## 3. Results

After inclusion and exclusion criteria were applied, fourteen studies [12,13,14,15,16,17,18,19,20,21,22,23,24,25], involving a total of 1527 eyes with microbial keratitis that underwent therapeutic keratoplasty were included in the qualitative analysis. Most articles were retrospective, except the study of Sourlis et al. [22], which provided a prospective approach (Table 2).

In six studies comprising 1038 (67.9%) patients, authors enrolled eyes with microbial keratitis of mixed etiologies [12,14,15,17,18,21], while others focused specifically on fungal [13,16,19,22,23] or Acanthamoeba keratitis [20,24,25]. The follow-up period varied from 6 to 24 months, with a weighted mean across studies of 17.25 months.

### 3.1. Etiological Spectrum of Microbial Keratitis Requiring Therapeutic Keratoplasty

A comprehensive etiological description was provided by most studies. Out of the total of 1527 patients, 55.8% (853 eyes) were positive for fungal keratitis, 16.9% (258 eyes) presented bacterial infection, while Acanthamoeba was found in 8.6% (130) cases. In fungal keratitis, *Fusarium* and *Aspergillus* spp. were predominant, especially in Asian cohorts [12,14,18]. Diabetes and history of vegetal matter trauma were the most frequent encountered risk factors [12,19,26].

Bacterial keratitis was less frequent, with an overall incidence varying from 14.3% [18] and 50% [14]. Chen [12] and Tew [21] found a predominance of Gram-negative bacteria, especially *Pseudomonas* spp., while Bajracharya [14] reported the most frequent cause as Streptoccocus (48%) and Staphyloccocus (28.8%). Non-tuberculous *Mycobacterium* were encountered in 29.2% of cases in the study of Chen et al. [12], being associated with previous corneal trauma with iron dust.

Acanthamoeba was a rare encounter overall, in retrospective studies with consecutive enrolling, varying from 0.5% [18] to 13.9% [12]. However, several case series focused selectively on this specific pathogen [20,24,25].

In twenty-seven cases (1.7%), bacteriological exam confirmed mixed infections, involving multiple pathogens. A substantial proportion of cases were culture-negative (258 eyes, 16.9%), ranging from 17.3% [13] to 70.2% [17], highlighting significant challenges in microbial diagnosis.

### 3.2. Surgical Technique and Postoperative Outcomes

While intensive targeted antimicrobial therapy remains the cornerstone of initial management for all types of microbial keratitis, the surgical intervention becomes necessary when medical treatment proves inefficient, when corneal ulcers progress to impending perforation, or when deep stromal involvement precludes healing, leading to scarring, perforation, endophthalmitis, and vision loss despite timely therapy [18,19,27,28]. The exact timing varied largely across the reviewed studies, depending both on the ophthalmologist’s decision in agreement with clinical evaluation of cases, but also on the availability of the grafts.

In the reviewed studies, most patients underwent TPK (89.1%), while small case series used DALK in selected cases [16,18,23], including less severe, fungal, bacterial, or Acanthamoeba keratitis.

The choice of procedure depends mainly on the extent of stromal involvement and endothelial status. The main indications for TPK include non-response to maximal medical therapy, impending or actual perforation, and deep stromal or limbal extension where medical treatment alone cannot ensure eradication of the infection, as consistently reported across the included studies [23].

On the contrary, DALK may be employed in anterior infections with preserved endothelium, ensuring a quicker recovery and fewer postoperative complications [16,20,23,24].

Therapeutic penetrating keratoplasty represents a crucial surgical option in the management of severe or refractory microbial keratitis. Its primary objectives are the eradication of infection and the restoration of corneal integrity, rather than visual rehabilitation. Thus, most authors stated that the corneal trephination of the host should exceed about 1.00 mm beyond the lesion to ensure clean margins. The donor button is generally 0.25–1.00 mm larger than the transplantation bed. Although anatomical restoration is generally successful following therapeutic penetrating keratoplasty, visual recovery and long-term rehabilitation remain limited, reflecting the challenges of achieving optical clarity in eyes that have undergone surgery in the setting of active infection and inflammation (Table 3).

In the reviewed studies, TPK was associated with an infection control rate varying between 69.2% and 100% and preservation of the globe in 84.6–100% in different subgroups. In studies reporting results of TPK for multiple etiologies, fungal keratitis was associated with poorer outcomes, compared to bacterial corneal ulcers (cure rate: 69.2–84.2% vs. 90.2–93.9%; anatomical success 77.2–87.8% vs. 95–95.5%). Better outcomes were described in fungal keratitis undergoing early TKP [15] and necessitating smaller (<8.00 mm) grafts [13].

However, the postoperative period was associated with a large number of complications, the most important being graft failure (29–87.7% at 12 months), higher in fungal infections. The major factor for primary graft failure in the first 30 days post-TKP was reinfection [12,14,18], while stromal and endothelial rejection affected graft survival in long-term follow-up.

Li et al. [13] investigated the correlations between the size of graft (larger than 8 mm) and the graft rejection. The authors found a significantly higher rate in cases with corneal graft of 8 mm or larger (43.75%) vs. 24%, stating as possible explanation the higher immune response, but also advanced corneal disease in these cases. Similar findings were reported by Raj et al. [17] and Zhang [18]. They found that all cases with postkeratoplasty glaucoma, cataract, and graft infection were encountered in cases that underwent TKP using a graft size > 8 mm. Hypopyon, corneal perforation, and delayed surgery were associated with worse outcomes [18].

In the reviewed studies, the outcomes of DALK in microbial keratitis were reported for small group studies, involving 10–27 cases [16,23,24]. This approach allows the removal of the diseased stroma while maintaining the healthy host endothelium, thereby reducing the risk of immunologic rejection and other endothelial-related complications [17]. Sabatino et al. [16] found optimal results for DALK in case of active fungal lesion involving optical zone, confined within 6.00 mm of central cornea, depth varying between 150 and 300 µm, as measured by AS-OCT, and poorly responsive to targeted medical therapy, but with no signs of inflammation in the anterior chamber. Alternatively, Wang et al. [23] used a two-step procedure, using amniotic flap to stabilize the epithelium and succeeded in a seria of ten cases with high-risk fungal keratitis, characterized by deep stromal infiltration, endothelial plaque, an/or hypopyon.

In Acanthamoeba keratitis, Qi et al. [24] found that DALK achieved better rate in infection control compared to TPK (91.7% vs. 88.9%). However, the authors acknowledge that TPK group included a higher proportion of patients with stage 3 compared to DALK group. Moreover, Bagga et al. [20] found better outcomes with DALK in mild-to-moderate AK patients, compared to severe cases, with lesions exceeding 8 mm.

### 3.3. Corneal Transplant in Microbial Keratitis-Etiology-Specific Considerations

The goals of TPK are the eradication of infection, restoration of globe integrity, and visual rehabilitation. Surgical planning and postoperative management vary with the causative organism. However, the available data are provided by retrospective series with heterogeneous methodologies, which may be a potential source of bias.

#### 3.3.1. Corneal Transplant in Fungal Keratitis

FK presents a considerable diagnostic and treatment challenge for ophthalmologists, and it is associated with significant ocular complications [1]. It typically develops after ocular trauma, where corneal lesions become contaminated with soil particles and plant matter. Moreover, it is suggested that the incidence of fungal keratitis has increased as a result of the extensive use of corticosteroids and broad-spectrum antibiotics [2].

Based on the depth of stromal infiltration, corneal ulcers are categorized as mild, moderate, or severe depending on the degree of stromal involvement. Mild ulcers are defined as those with less than one-third superficial stromal involvement. Moderate ulcers involve one- to two-thirds of the stromal thickness, while severe ulcers extend beyond two-thirds of the stroma, may reach the posterior layers or approach the limbus, and are often associated with impending or actual perforation, requiring TPK [1].

Medical therapy remains the cornerstone of initial management in fungal keratitis, with the main goal of eradicating the infection while preserving the structural integrity of the cornea. The depth of stromal infiltration plays a decisive role in guiding therapy and predicting clinical outcomes in fungal keratitis, the posterior stromal involvement considered to be a key predictor of medical failure [1,3].

Antifungal management in fungal keratitis is largely influenced by the underlying etiologic agent, as different fungal species exhibit variable drug susceptibility and clinical behavior. Natamycin remains the drug of choice for superficial fungal keratitis, demonstrating excellent efficacy in ulcers limited to the anterior stroma [4,22]. Deeper stromal involvement often necessitates the addition of azoles such as itraconazole or topical amphotericin B to enhance antifungal coverage [28]. Systemic antifungal therapy, with oral itraconazole (200 mg once daily), is indicated in patients presenting with impending or actual perforation, dense hypopyon, or inadequate response to topical agents [1]. Also, in severe cases of fungal keratitis, particularly those with intraocular involvement, additional systemic agents such as the allylamine terbinafine hydrochloride (250 mg daily) and oral voriconazole (200 mg twice daily) have been used to broaden antifungal coverage and achieve effective intraocular concentrations [5]. 

For Candida and Curvularia infections, Sabatino et al. [16] reported the addition of topical amphotericin B 0.15% to the standard regimen (1% voriconazole eye drops and 200 mg/day voriconazole tablets) [6].

In severe cases, Sourlis et al. [22] highlighted the potential benefit of early intracameral administration of polyene antifungals. By delivering the drug directly into the anterior chamber and repeating the injection when necessary, this approach allows for higher local antifungal concentrations while minimizing systemic toxicity. Such targeted therapy may enhance infection control in cases with deep stromal or intraocular extension, where topical and systemic treatments alone are often insufficient [5].

Intrastromal antifungal injections have also been utilized as an adjunctive measure before keratoplasty in cases of deep fungal keratitis [23,28,29]. In this context, Wang et al. [23] described a staged surgical approach in which intrastromal voriconazole injections (0.1 mL, 100 μg/0.1 mL) were administered circumferentially around the infiltrate prior to keratoplasty. This technique was intended to locally reduce the fungal load and control the infection, thereby allowing a safer and more effective lamellar transplantation to be performed once corneal inflammation had subsided [23].

The importance of surgical timing has been particularly emphasized in the context of fungal keratitis. Sourlis et al. [22] highlighted that performing a large-diameter TPK early in the disease course can enhance anatomical control and reduce the risk of globe loss in severe fungal ulcers. However, in many settings, the timing of surgery is constrained by the limited availability of donor corneas, which can delay intervention and compromise both anatomical and visual outcomes. Decreased infection load prior to surgery, without unnecessary delays that may lead to extensive damage of the corneal tissue, should be taken into account [25]. Consequently, early recognition of infection and prompt initiation of appropriate therapy remain essential to improving the prognosis of fungal keratitis [1].

**Figure 2:**
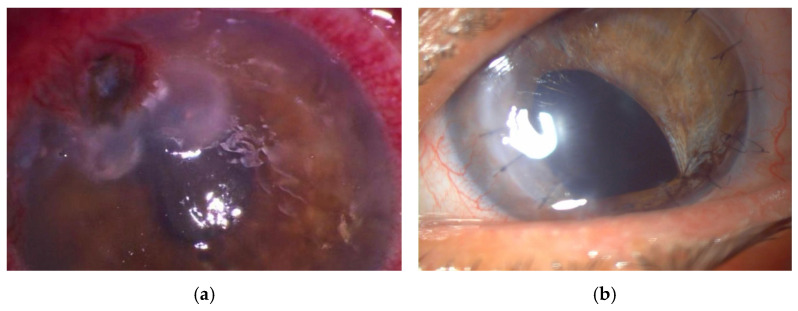
Advanced fungal keratitis (magnification ×16): (**a**) preoperative image showing a dense corneal infiltrate with marked epithelial defect; (**b**) postoperative aspect at 1 month following TPK: clear graft with mild interface haze.

TPK in fungal keratitis is associated with a higher risk of recurrences and graft melt and failure, compared to bacterial etiology. Larger grafts (≥1 mm beyond infiltrate) should be used because fungi extend beyond visible margins. During surgery, a careful removal of all plaques, endothelial exudates, hypopyon, and involved iris, if necessary, was performed in accordance with previous reports [13,19,23] and is illustrated in Figure 2.

Systemic and topical antifungal therapy should be continued for weeks in the postoperative period. A key postoperative challenge is determining the timing of topical corticosteroid. While topical corticosteroids are indispensable for preventing graft rejection, premature initiation risks reactivation of infection [22,23]. Conversely, excessive delay can lead to anterior segment disorganization, synechiae, and neovascularization, which may compromise both the therapeutic graft and the potential for future optical keratoplasty [2,5]. Mundra et al. [19] initiated topical corticosteroids 10–14 days postoperatively in the absence of infection recurrence, balancing infection control with the need to protect the graft [2]. Similarly, Sourlis et al. [22] delayed the initiation of corticosteroids for one to two weeks after surgery, and as an interim measure, systemic cyclosporine A was used to provide immunosuppression during the early postoperative period.

Most studies emphasize that therapeutic keratoplasty performed during active infection primarily aims to preserve globe integrity rather than restore vision [13]. However, postoperative visual outcomes remain modest in the majority of cases. Mundra et al. reported that, although anatomical restoration was generally successful, visual rehabilitation was often poor, partly because corticosteroids cannot be started immediately after surgery, leading in some cases to anterior segment disorganization and limited potential for future keratoplasty [2]. Sourlis et al. [22] reported favorable outcomes in eyes that did not require further surgical intervention, with a mean corrected distance visual acuity 0.96 ± 1.17 Log MAR at end of follow-up period.

Complications following keratoplasty in fungal keratitis remain a significant concern and vary across studies. In the large series by Mundra et al. [19], recurrent infection occurred in 10.1% (20/198); importantly, postoperative corneal edema was documented in 71 eyes among 130 clear grafts (≈54.6%), and 27/71 (≈38.0%) of those edematous grafts required a repeat keratoplasty, highlighting postoperative edema as an important cause for subsequent re-keratoplasty [2]. In a separate cohort focused on severe disease, Sourlis et al. [22] reported repeated TPK in 33.3% (9/27), most commonly for recurrent infection (25.9%, 7/27) and less often for graft failure (7.4%, 2/27). Li et al. [13] found that larger trephinations (≥8.0 mm) carried higher complication rates than smaller ones: graft rejection 43.8% (28/62) vs. 24.0% (12/50) and secondary glaucoma 16.1% (10/62) vs. 4.0% (2/50), despite no significant differences in postoperative vision, graft clarity, or infection recurrence between size groups.

Postoperative complications following therapeutic keratoplasty for fungal keratitis remain a major challenge and require individualized management to preserve the globe and, where possible, restore vision. The most frequent and clinically significant complication is recurrence of fungal infection, which requires prompt and targeted management. Mundra et al. [19] reported that recurrence occurred in 10.1% of cases and was managed either medically, with antifungal therapy, or through surgical intervention required in cases with intraocular extension, such as anterior chamber lavage with amphotericin B or pars plana vitrectomy combined with intravitreal antifungal injections [2,19]. Several eyes required multiple therapeutic keratoplasties because of recurrent infection and graft failure, reflecting the difficulty of achieving long-term disease control in severe fungal keratitis, leading in a small number of cases to evisceration or enucleation [13,19,22].

An additional postoperative challenge is the management of secondary glaucoma, which may arise from elevated intraocular pressure after therapeutic keratoplasty, an issue further compounded by the fact that reliable IOP assessment is both difficult and essential in the follow-up of patients who have undergone penetrating keratoplasty [13]. Li et al. [13] reported that treatment involved a stepwise approach, beginning with antiglaucomatous medication and progressing to surgical intervention in refractory cases. Procedures such as trabeculectomy or combined trabeculectomy with cyclodistructive techniques were employed to achieve pressure control, maintain graft clarity, and preserve visual potential [13,19,22].

However, outcomes after DALK for FK have been consistently favorable, reflecting the potential of this technique to achieve both infection eradication and visual rehabilitation when applied in appropriate cases [16,23].

#### 3.3.2. Corneal Transplantation in Bacterial Keratitis

**Figure 3:**
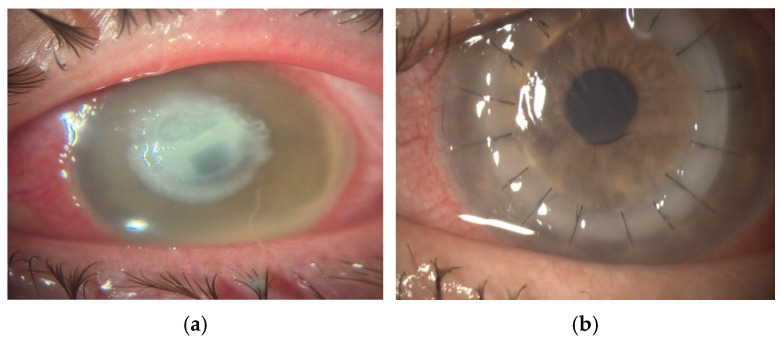
Pseudomonas aeruginosa keratitis (magnification ×16): (**a**) preoperative aspect: central epithelial defect, dense central stromal infiltrate, and hypopyon; (**b**) postoperative aspect one week following early TPK: well-centered graft, fine Descemet folds, and mild peripheral edema.

Bacterial keratitis is the most frequent form of microbial keratitis and represents a true ophthalmic emergency due to its potential for rapid corneal destruction if not treated promptly. A history of contact lens wear is the most significant predisposing factor, making it essential to inquire about lens use and hygiene practices in any patient presenting with features suggestive of infectious keratitis [30]. Clinically, bacterial ulcers typically present as a round epithelial defect associated with a dense, well-demarcated white stromal infiltrate, and in more severe cases, they may be accompanied by a hypopyon [31], as shown in Figure 3. Intensive topical antibiotics (fortified aminoglycosides, cephalosporins, or fluoroquinolones) remain the cornerstone of treatment, often supplemented with systemic therapy in severe or deep infections [30,32]. The success of medical therapy in bacterial keratitis largely depends on the rapidity of diagnosis, the depth and extent of stromal involvement, and the virulence of the causative organism. Corneal transplantation should be considered in bacterial keratitis when medical therapy fails to control the infection or when there is an imminent risk of corneal perforation, after a reasonable disclosure and obtaining the informed consent [12,14,33] (Figure 3).

In the reviewed studies, topical steroids were initiated relatively early (after epithelialization and clear clinical control, usually 3–5 days following TPK for bacterial keratitis), reflecting the greater safety of early immunomodulation once the infectious load has been surgically removed and underscoring the contrast with the more cautious steroid protocols required in fungal keratitis [12,14,17].

In the series by Tew et al. [21], therapeutic penetrating keratoplasty achieved high rates of infection eradication in bacterial keratitis (91.9%), yet graft clarity declined substantially over time, with only 38.6% of grafts remaining clear at one year. Graft diameter emerged as an important prognostic factor, as smaller grafts (<8.5 mm) demonstrated significantly better one-year survival compared with larger trephinations (>8.5 mm) (46.2% vs. 22.2%, *p* = 0.029) [21]. Similarly, in the cohort reported by Chen et al. [12], therapeutic PKP achieved infection eradication in the vast majority of bacterial keratitis cases (37/41), with 85% of grafts remaining clear at one month and approximately two-thirds maintaining clarity at one year. Graft diameter was a key prognostic factor: smaller trephinations (≤8.5 mm) demonstrated notably better one-year clarity (18/24) compared with larger grafts (>9.5 mm), of which only one-third remained clear at one year [12]. Overall, these findings highlight the heterogeneity of clinical presentations and the need for individualized surgical strategies in the management of bacterial keratitis.

#### 3.3.3. Corneal Transplant in Acanthamoeba Keratitis

Acanthamoeba keratitis (AK) is an uncommon but sight-threatening corneal infection caused by free-living amoebae of the genus *Acanthamoeba*. Its epidemiology varies across regions. In developed countries, contact lens wear represents the predominant risk factor, whereas in Asian countries, AK is more commonly associated with ocular trauma and exposure to contaminated water, particularly among agricultural workers [12,18].

Several authors have attempted to classify AK according to clinical severity, mainly to guide prognosis and surgical decision-making. Qi et al. [24,34,35,36,37,38] described a clinical staging system based on disease progression. Stage 1 was characterized by worsening stromal edema and increasing infiltrate density; stage 2 involved non-healing ulcers that tended to enlarge with a risk of perforation; and stage 3 represented the most advanced form, defined by the development and progression of hypopyon.

Importantly, clinical severity can be exacerbated by missed or delayed diagnosis, due to inconsistent signs in early stages, which may be easily confounded with contact lens-related irritation and bacterial or viral keratitis. Steroids suppress local immune response, leading to delay cyst clearance, accelerate trophozoite growth, and are associated with deeper stromal invasion and the risk of bacterial coinfection [34,35,36]. Wouters et al. (2022) demonstrated that prior topical corticosteroid use was associated with a significantly longer diagnostic delay (62 ± 62 days vs. 23 ± 39 days in the non-steroid group; *p* < 0.001), potentially allowing the disease to progress into more advanced stages before appropriate therapy was initiated [36]. Moreover, a comprehensive study of Scruggs et al. [34] found that corticosteroid use prior to AK diagnosis led to significant delays in diagnosis and poorer outcomes.

Medical therapy remains the first-line approach in Acanthamoeba keratitis and relies primarily on the use of biguanides and diamidines because of their activity against both the trophozoite and cyst forms of the organism [37,38,39,40,41,42,43]. In the series by Bagga et al. [20], all cases of microbiologically confirmed AK were treated according to an institutional protocol consisting of topical polyhexamethylene biguanide (PHMB) 0.02% combined with chlorhexidine 0.02%. These agents were not commercially available and were prepared in the microbiology laboratory by diluting 20% stock solution with carboxymethylcellulose eye drops to the required concentration [34,35,36,37,38,39,40]. Similarly, Qi et al. [24] reported initiating intensive medical therapy immediately after diagnosis, with topical chlorhexidine 0.02% and PHMB 0.02% administered every 30 min, along with metronidazole eye drops applied hourly. This aggressive dosing regimen reflects the need to achieve adequate penetration and sustained antimicrobial activity against both trophozoite and cyst forms of Acanthamoeba, particularly in the early stages of treatment.

However, up to 40% of cases remain unresponsive to targeted therapy and require surgery [33]. Diagnostic delays lead to more advanced disease, greater tissue damage, higher rates of therapeutic keratoplasty, and poorer visual prognosis.

Progression of infection, manifested as an increase in infiltrate size or progressive stromal thinning despite appropriate therapy, was considered treatment failure and prompted surgical intervention, either in the form of DALK or PK depending on the depth of corneal involvement [20,24]. Due to its specific pattern, characterized by perineural and deep stromal spread, larger grafts are usually required, considering that the disease spread surpasses the clinical visible lesion.

Early intervention is recommended when medical treatment fails, with smaller graft sizes (<8.5 mm) reporting better cure rates (86.7%) [36]. These findings underline that the timing of keratoplasty is largely determined by the lack of response to medical therapy rather than by a predefined temporal threshold. In the study by Qi et al. [24], DALK was considered when corneal infiltrates involved more than four-fifths of the stromal thickness but did not extend through the entire cornea. This decision was based on slit-lamp examination and AS-OCT findings, and surgery proceeded once Descemet’s membrane was exposed. If, during BB-DALK, endothelial plaques were identified or perforation was present, the procedure was converted to PK [24]. Postoperative use of topical corticosteroids in Acanthamoeba keratitis requires careful timing to balance the risk of graft rejection with the danger of reactivating infection. The timing varied between 1 week and 2 months after surgery in the reviewed studies [20,24,25], in the context of no clinical suspicion of residual infection, alongside with anti-amoebic therapy, to minimize the risk of recurrence. The dosage of topical corticosteroids was subsequently adjusted only if the ocular surface remained stable and no recurrence was detected.

Outcomes after keratoplasty in Acanthamoeba keratitis vary considerably depending on disease severity and surgical technique. Key prognostic factors include older age, delayed diagnosis, corticosteroid use before prompt diagnosis, poor initial best corrected visual acuity (BCVA), and AK stage at presentation [20,24,25,36]. Advanced cases are more prone to recurrence and graft failure, while less severe infections managed with lamellar techniques such as BB-DALK achieve higher graft survival, lower rates of immune rejection, and better visual rehabilitation [24]. Ultimately, meticulous surgical planning, appropriate patient selection, and vigilant postoperative follow-up are crucial to improving both anatomical stability and long-term visual function in this challenging form of infectious keratitis.

## 4. Discussion

Infectious keratitis represents a major global cause of vision loss and can severely compromise ocular integrity if not promptly and adequately treated. The heterogeneity of this condition, its unpredictable clinical course, variability in the timing of treatment initiation, and the frequent inability to accurately identify the causative pathogen represent major therapeutic challenges and limitations in the development of a clear treatment algorithm [44]. The primary and definitive goal is eradication of the infection. Whether managed medically or surgically, therapeutic success, ocular preservation, and visual recovery ultimately depend on effective control of the underlying infectious process. These infections, either fungal, bacterial, or Acanthamoeba, though microbiologically distinct, share the following overlapping clinical challenges: rapid corneal destruction, risk of perforation, and variable response to medical therapy. Across all types, early diagnosis and organism-specific treatment are critical determinants of outcome, often defining whether surgical intervention can be deferred or avoided [12,22,24]. Nevertheless, even with optimal medical management, progression to therapeutic keratoplasty remains frequent, underscoring the importance of timely identification of refractory cases and individualized surgical planning.

Optimal timing of the surgical procedure still remains a challenging subject. Both conservative and surgical overtreatment should be avoided [25]. Corneal transplant in microbial keratitis is effective in infection cure, but it is associated with high morbidity and poor visual outcomes in long-term follow-up series, so a judicious appraisal of the cases which will not respond to conservative approach is needed. On the other hand, it is important to avoid delaying keratoplasty in patients with advanced disease [25]. Across etiologies, better outcomes are associated with early presentation, superficial infiltrates, and absence of hypopyon, whereas advanced stromal disease, large lesions, and endothelial involvement carry higher risks of recurrence, graft failure, and poor visual prognosis [13,15,24].

**Figure 4:**
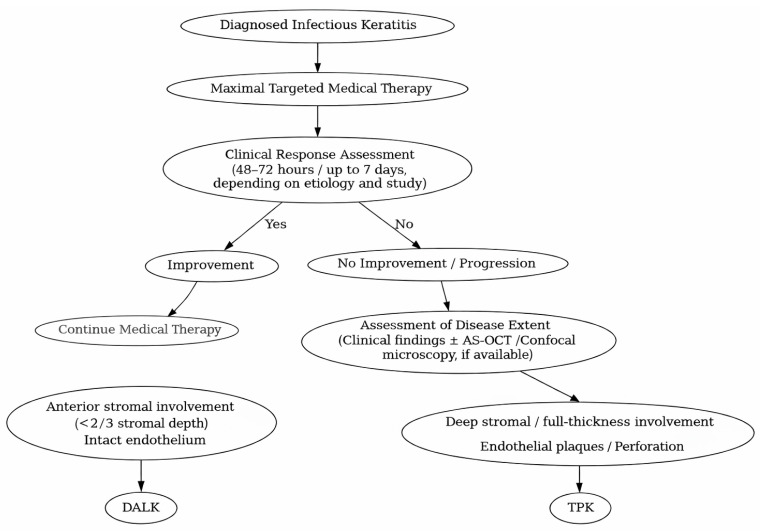
Decision-making algorithm for the management of infectious keratitis.

The size of the lesion is the key determinant of the graft size. Larger grafts, closer to corneal limbus, were associated with higher rate of immunological rejection [13,20]. Moreover, deeper lesions, involving endothelium and inner third of the stroma which require TKP, exhibited poorer outcomes and increased number of complications, compared to the less invasive DALK [16,18,24,45]. A simplified decision-making algorithm is presented in Figure 4.

Across etiologies, the outcomes summarized in this review consistently demonstrate a divergence between anatomical and functional success. Long-term outcomes following keratoplasty for infectious keratitis are influenced by a multifactorial interplay that includes the causative organism, the extent of stromal involvement, the timing of intervention, and the surgical technique employed [46,47,48].

Therapeutic keratoplasty, whether performed as TPK or selected lamellar approaches, achieves high rates of infection control and globe preservation, which represent the primary and clinically meaningful endpoints in advanced infectious keratitis. Bacterial etiology was associated with more favorable outcomes, compared to fungal and Acanthamoeba infections. However, visual recovery frequently remains modest, particularly in eyes undergoing urgent surgery during severe inflammation. This gap likely reflects the cumulative burden of preoperative disease severity (deep stromal necrosis, scarring, and limbal involvement), the active inflammatory state at the time of surgery, and postoperative constraints on immunosuppression, especially in fungal and Acanthamoeba keratitis where corticosteroids must be introduced cautiously and often delayed. Advanced disease has been associated with lower recurrence-free graft survival, likely reflecting a higher inflammatory burden and deeper stromal involvement at the time of intervention. In this context, lamellar techniques such as BB-DALK appear to confer immunological advantages when the endothelium is spared, resulting in improved graft survival and a markedly lower risk of immune rejection compared with TKP. On the other hand, in cases of advanced fungal keratitis, complete excision of infected tissue often necessitates larger trephinations. While such extensive grafts may be surgically required to achieve adequate infection control, larger trephinations have been associated with higher rates of graft rejection and secondary glaucoma, highlighting the balance between achieving adequate infection control and the risk of postoperative complications in advanced fungal disease [49].

Although large-diameter grafts are associated with higher rates of postoperative complications, timely surgical intervention may nevertheless offer important anatomical benefits in advanced fungal keratitis. In particular, Sourlis et al. highlighted that performing a large-diameter TPK early in the disease course can improve anatomical control and reduce the risk of globe loss in these cases [22].

### Limitations of Current Evidence and Future Directions

This review has some limitations, due to the heterogenicity and the lack of high-level evidence reports. The available evidence on corneal transplantation in infectious keratitis is limited by small, predominantly retrospective single-center studies with variable follow-up durations and heterogeneous treatment protocols, making inter-study comparisons difficult. Surgical indications, timing of intervention, graft size selection, and postoperative corticosteroid regimens are not standardized, and outcome measures such as graft survival, recurrence, and visual rehabilitation are reported inconsistently. A comparative appraisal of the outcomes following PKP and DALK remains limited, the results being reported by observational cohorts rather than controlled trials. Moreover, the severity of infection was lower in DALK subgroups, representing a cause of potential bias.

Future research should prioritize standardized reporting, prospective multicenter studies, and clearer criteria for surgical timing and postoperative immunomodulation, supported by objective diagnostic tools such as confocal microscopy and anterior segment OCT.

Beyond conventional medical and surgical management, increasing attention has been directed toward emerging therapeutic strategies aimed at addressing current limitations in the treatment of infectious keratitis. These include the exploration of novel adjunctive and alternative antimicrobial approaches, the growing challenge of antifungal resistance, and advances in tissue engineering-based corneal reconstruction [27,50,51]. Together, these developments reflect ongoing efforts to improve infection control, preserve corneal integrity, and expand future options for visual rehabilitation in severe or refractory cases [27,52].

Photoactivated chromophore for infectious keratitis corneal cross-linking (PACK-CXL) combines topical riboflavin with ultraviolet-A irradiation to exert both antimicrobial and biomechanical effects, including inhibition of microbial replication, damage to pathogen cell walls, and increased stromal resistance to enzymatic degradation [53]. In a study by Said et al. [53], adjunctive PACK-CXL was shown to halt corneal melting and prevent corneal perforation in 100% of treated patients, compared with 84% in the non-CXL group. In another study, a success rate of 90.5% was reported at the third postoperative month, with statistical analyses demonstrating a significant reduction in corneal ulcer size when PACK-CXL was used as an adjunct to standard antimicrobial therapy (*p* = 0.031). PACK-CXL may represent a promising adjunctive therapeutic option, with potential benefits including improved infection control independent of antimicrobial resistance, stabilization of corneal melting, and reduced need for emergency keratoplasty [54].

A systematic review and meta-analysis conducted by Ting et al. [55] found that early adjuvant AMT may accelerate corneal healing and improve visual outcomes in patients with moderate-to-severe bacterial and fungal keratitis. However, the authors emphasized that further adequately powered and well-designed randomized controlled trials are required to better define the role of adjuvant AMT in active infectious keratitis, particularly in Acanthamoeba keratitis [54].

The increasing incidence of antimicrobial resistance among ocular pathogens has stimulated interest in alternative therapeutic strategies beyond conventional antibiotics and antifungal agents. In this context, designed host defense peptides (dHDPs) have emerged as a promising approach, representing synthetic analogs of endogenous host defense peptides engineered to optimize antimicrobial efficacy against pathogenic microorganisms. In a study by Clemens et al. [55], the topically applied antimicrobial peptide RP444 demonstrated rapid bactericidal activity, broad-spectrum efficacy, selective targeting of bacterial cell membranes, and a reduced probability for the development of bacterial resistance. These findings support further preclinical evaluation of RP444 as a potential therapeutic candidate for the treatment of sight-threatening bacterial keratitis [55].

Recent post hoc analyses of large fungal keratitis cohorts from South India have identified a concerning trend toward reduced susceptibility of corneal fungal isolates to commonly used antifungal agents, including natamycin and voriconazole, over the past decade. This pattern, particularly pronounced among filamentous fungi such as *Fusarium* and *Aspergillus*, underscores potential challenges in medical management. However, further studies are required to confirm these findings and to determine their generalizability to other geographic regions [56].

Another innovative approach currently under investigation is tissue engineering-based corneal reconstruction, which seeks to address the limited availability of optical-grade donor corneas by developing partial- or full-thickness bioengineered replacements for diseased corneal tissue. Such constructs have the potential to eliminate the need for donor screening, enable large-scale manufacturing and distribution, and allow customization according to individual patient requirements. An ideal bioengineered corneal graft should fulfill several essential criteria. Functionally, it must be optically transparent and possess an appropriate refractive index to ensure accurate image formation on the retina. Mechanically, the graft should provide sufficient structural strength to protect intraocular tissues while remaining permeable to nutrients and oxygen to support corneal cells survival. From a biomimetic standpoint, successful integration depends on host stromal cell migration and colonization within the implant. Finally, the graft should be non-antigenic and non-inflammatory to avoid immune-mediated vascularization and opacification [57].

## 5. Conclusions

Therapeutic corneal transplantation may be globe-saving procedure in unresponsive microbial keratitis being associated with high rate of infection control, but poor functional results. Postoperative outcomes are strongly influenced by the type of organism, timing of surgery, and adequacy of medical therapy. DALK is a less invasive option for microbial keratitis confined to the anterior/mid stroma, associated with higher graft survival rate and improved vision. Further studies are needed to better define the surgical criteria, postoperative immunomodulation, and to optimize long-term outcomes.

## Figures and Tables

**Table 1:** Quality appraisal of included studies on therapeutic keratoplasty for microbial keratitis.

Author (Year)	Study Design	Quality Appraisal Tool	Score/Rating
Chen WL et al., 2004 [12]	Case series, retrospective	JBI	7/10 Moderate
Li C et al., 2012 [13]	Cohort, retrospective	NOS	8/9 High
Bajracharya, 2015 [14]	Case series, retrospective	JBI	7/10 Moderate
Koçluk, 2017 [15]	Case series, retrospective	JBI	7/10 Moderate
Sabatino et al., 2017 [16]	Case series, retrospective	JBI	8/10 High
Raj A et al., 2018 [17]	Case series, retrospective	JBI	7/10 Moderate
Zhang Q et al., 2019 [18]	Cohort study, retrospective	NOS	8/9 High
Mundra J et al., 2019 [19]	Case series, retrospective	JBI	9/10 High
Bagga B et al., 2020 [20]	Case series, retrospective	JBI	8/10 High
Tew TB et al., 2020 [21]	Case series, retrospective	JBI	8/10 High
Sourlis C et al., 2022 [22]	Case series, prospective	JBI	8/10 High
Wang YC et al., 2023 [23]	Case series, retrospective	JBI	7/10 Moderate
Qi X et al., 2024 [24]	Cohort study, retrospective	NOS	9/9 High
Abu Dail Y et al., 2024 [25]	Cohort study, retrospective	NOS	8/9 High

**Table 2:** Summary of included studies on therapeutic keratoplasty for microbial keratitis.

Author (Year)	Country	Sample Size (Eyes)	Type of Keratitis (%)	Surgical Procedure	Follow-Up (Months)	Main Outcomes
Chen WL et al., 2004 [12]	Taiwan	108	Fungal (48.1%), bacterial (40%), Acanthamoeba (13.9%)	TPK	12	Infection control, graft clarity; anatomical success
Li C et al., 2012 [13]	China	116 (64 graft ≥ 8 mm; 52 graft < 8 mm)	Fungal	TPK	24	Graft diameter effect on infection control, graft rejection, graft clarity, visual acuity
Bajracharya, 2015 [14]	Nepal	180	Bacterial (28%), fungal (28%), unknown (44%)	TPK	29 ± 23	Infection control, graft clarity, anatomical success, visual acuity
Koçluk, 2017 [15]	Turkey	25 (13 early group; 12 delayed group)	Bacterial (36%), fungal (28%), mixed (24%)	TPK	11.2 ± 3.9	Early vs. late surgery outcomes
Sabatino et al., 2017 [16]	Italy	23	Fungal	Early DALK (17 BB-DALK; 6-manua l dissection)	32 ± 10	Infection control; graft transparency, anatomic success, visual acuity
Raj A et al., 2018 [17]	India	57	Bacterial (8.8%), fungal (19.3%), viral (1.8%), unknown (70.2%)	TPK	13 ± 4.7	Infection control; graft transparency, anatomic success, visual acuity
Zhang Q et al., 2019 [18]	China	561	Bacterial (14.3%), Fungal (56.5%), Acanthamoeba (0.5%), mixed (2.7%), unknown (26%)	TPK (80.9%); DALK (19.1%)	21 ± 13.5	Anatomical success; infection control
Mundra J et al., 2019 [19]	India	198	Fungal	TPK	24 ± 17	Infection control; graft transparency, anatomic success, visual acuity
Bagga B et al., 2020 [20]	India	23	Acanthamoeba	DALK	6–12	Infection control, graft survival
Tew TB et al., 2020 [21]	Taiwan	107	Bacterial (57.9%), fungal (38.3%), Acanthamoeba (9.3%), mixed (5.6%)	TPK	5–12	Infection control, anatomic success, graft survival
Sourlis C et al., 2022 [22]	Germany	40	Fungal (100%)	Early TPK	14.9 ± 17.6	Infection control
Wang YC et al., 2023 [23]	China	10	Fungal (100%)	Two-step: flap + DALK	9.25 ± 3.39	Graft survival, infection control, visual acuity
Qi X et al., 2024 [24]	China	27	Acanthamoeba	BB-DALK vs. TPK	12–36	Graft survival, infection control, visual acuity
Abu Dail Y et al., 2024 [25]	Germany	28	Acanthamoeba	Early low-load TKP	53 ± 42	Graft survival, infection control, visual acuity

**Table 3:** Outcomes, visual acuity, and complications of therapeutic keratoplasty.

Study	Cure Rate (%)	Anatomical Success (%)	Graft Clarity (%) 1 Month	Final Graft Clarity (%)	Graft Failure (%) at Final Evaluation	Visual Acuity in Clear Grafts
Chen WL et al., 2004 [12]	79.6% (90.2%—bacterial; 69.2% fungal; 86.7% Acanthamoeba)	89.8% (95.1%—bacterial; 84.6% fungal; 93.3% Acanthamoeba)	75.9% (85.4%—bacterial; 65.4% fungal; 86.7% Acanthamoeba)	62.4% (68.8%—bacterial; 51.3% fungal; 78.6% Acanthamoeba)	43.5% (31.3% bacterial; 48.7% fungal; 21.4% Acanthamoeba)	>20/60 at 12 months: 37.7% (34.4% bacterial; 25% fungal; 63.6% Acanthamoeba)
Li C et al., 2012 [13]	87.5% vs. 91.4%, (larger vs. smaller grafts)	96.8% vs. 96.1% (larger vs. smaller grafts);	No info	67.7% vs. 80% (larger vs. smaller grafts)	43.75% vs. 24% (larger vs. smaller grafts)	>0.1 at 24 months: 20.96% vs. 20% (larger vs. smaller grafts)
Bajracharya, 2015 [14]	88.8% (73.5% fungal vs. 93.9% bacterial)	89.5% (77.2% fungal vs. 95.5% bacterial)	No info	37.2% (26.4% fungal; 32.5% bacterial; 47.6% culture-negative)	38.6%—endothelial failure; 24%—late infective keratitis	>6/60 at final follow-up: 25.4%
Koçluk, 2017 [15]	100% (early group); 83.7% (delayed group)	100% (early group); 83.7% (delayed group)	100% (early group); 83.7% (delayed group)	100% (early group); 83.7% (delayed group)	No info	Mean VA in early vs. delayed group: 0.4 vs. 0.1
Sabatino et al., 2017 [16]	100%	100%	No info	100%	None	Median VA 0.1 log MAR
Raj A et al., 2018 [17]	82.5%	85.9%	No info	70.1%	52.6%	>20/200 in 38.58%
Zhang Q et al., 2019 [18]	85.6%	87.7% (95% bacterial; 84.2% fungal; 66.7% Acanthamoeba)	No info	No info	29%	No info
Mundra J et al., 2019 [19]	89.9%	97%	73%	12.3%	87.7%	Mean VA at 12 months: 20/40
Bagga B et al., 2020 [20]	80% (advanced keratitis) vs. 92.3% (mild to moderate keratitis)	No info	No info	40% (advanced keratitis) vs. 84.6% (mild/moderate keratitis)	60% (advance keratitis > 8 mm) vs. 15.4% (mild/moderate keratitis)	Mean VA of 1.79 Log MAR
Tew TB et al., 2020 [21]	87% (bacterial 91.9%; fungal 80.5%; Acanthamoeba 90%)	93.5% (bacterial 95.2%; fungal 87.8%; Acanthamoeba 90%)	74.8% (bacterial 77.4%; fungal 73.2%; Acanthamoeba 70%)	46.5% (bacterial 38.6%; fungal 57.9%; Acanthamoeba 50%)	No info	No info
Sourlis C et al., 2022 [22]	82.5%	92.5%	No info	No info	66.4%	0.96 ± 1.17 Log MAR
Wang YC et al., 2023 [23]	100%	100%	No info	No info	No info	0.38 ± 0.14 Log MAR
Qi X et al., 2024 [24]	91.7% (BBDALK) vs. 88.9% (TKP)	100%	No info	No info	11% (no difference among subgroups)	0.71 ± 0.64 Log MAR
Abu Dail Y et al., 2024 [25]	96%	100%	No info	No info	30%	0.5 ± 0.6 Log MAR

## Data Availability

No new data were created or analyzed in this study.

## Supplementary Materials

The following supporting information can be downloaded at https://www.mdpi.com/article/10.3390/jcm15020871/s1, PRISMA checklist.

## Abbreviations

The following abbreviations are used in this manuscript:

AC	anterior chamber
ADK	advanced disease keratitis
AK	Acanthamoeba keratitis
AS-OCT	anterior segment optical coherence tomography
BB-DALK	big-bubble DALK
BCVA	best-corrected visual acuity
CDVA	corrected distance visual acuity
CF	counting fingers
CFCS	conjunctival flap covering surgery
CS	corticosteroids
CSP A	cyclosporin A
DALK	deep anterior lamellar keratoplasty
DMD	Descemet’s membrane detachment
FK	fungal keratitis
HM	hand movement
IV	intravenously
IVCM	in vivo confocal microscopy
LP	light perception
LSK	less severe keratitis
M	month/s
OPK	optical penetrating keratoplasty
PBK	pseudophakic bullous keratopathy
PHMB	polyhexamethylene biguanide
PKP	penetrating keratoplasty
PLK	posterior lamellar keratoplasty
PPV	pars plana vitrectomy
TPK	therapeutic penetrating keratoplasty
VCZ	voriconazole
